# Bacteriophage-mediated reduction of *Pseudomonas aeruginosa* biofilm on titanium surfaces for biomedical applications

**DOI:** 10.1007/s10096-026-05462-z

**Published:** 2026-04-08

**Authors:** Amanda Kelly Ferreira Sousa, Viviani Tadioto, Anderson Giehl, Helena Yurevna Caio, Estevão Brasiliense de Souza, Evelin Padilha Corrêa, Mariana Alves Elois, Estevan Rodrigues dos Santos Neto, Sérgio Luiz Alves Junior, Ariadne Cristiane Cabral da Cruz, Mara Cristina Scheffer, Maria Célia da Silva Lanna, Gislaine Fongaro

**Affiliations:** 1 Department of Microbiology, Immunology and Parasitology, Federal University of Santa Catarina (UFSC), Florianópolis, SC 88034-000 Brazil; 2 Department of Biological Sciences, Federal University of Ouro Preto (UFOP), Ouro Preto, MG 35400-000 Brazil; 3 Laboratory of Yeast Biochemistry (LabBioLev), Federal University of Fronteira Sul, Campus (UFFS), Chapecó, SC Brazil; 4 Department of Dentistry, Federal University of Santa Catarina (UFSC), Florianópolis, SC 88040-900 Brazil; 5 Division of Clinical Analyses of the University Hospital of the Federal University of Santa Catarina (HU/UFSC), Florianópolis, Brazil

**Keywords:** Biocontrol, Bacterial, Bacteriophages, Infection control, Medical devices

## Abstract

Biofilm-associated infections on biomedical devices represent a major clinical challenge due to their high tolerance to antimicrobial treatments and their association with chronic and recurrent infections. *Pseudomonas aeruginosa* is a clinically relevant biofilm-forming pathogen, particularly on titanium-based implants used in medical and dental applications.In this study, we quantitatively evaluated the antibiofilm activity of bacteriophages (phages) against *P. aeruginosa* biofilms on titanium by assessing reductions in viable cell counts and biofilm biomass. The phages were tested both for their ability to eradicate mature biofilms and to prevent the formation of new ones. Phage treatment resulted in a marked reduction of biofilm biomass and viable cells(reductions exceeding 90%). . For the ATCC strain, reduction efficiencies ranged from 50.53% to 99.78%, while for the clinical isolate, reductions varied from 0.01% to 99.94%. Phages PA4 and PA5 showed the highest and most consistent activity, achieving reductions above 99%. Prophylactic application effectively inhibited biofilm formation. No clear dose–response relationship was observed, and efficacy varied depending on strain, phage, and infection conditions. Phages show strong potential as antibiofilm agents on titanium surfaces. The observed variability highlights the complexity of phage–biofilm interactions and supports further in vivo studies and the development of tailored phage-based strategies for device-associated infections.

## Introduction

Biofilms are structured microbial communities that adhere to surfaces and are embedded in a self-produced extracellular polymeric matrix, representing one of the most challenging forms of infection in clinical and dental settings. Biofilm-associated infections on medical and dental devices are particularly problematic due to their high tolerance to antimicrobial therapies and their frequent association with chronic and recurrent infections, leading to increased morbidity, prolonged treatments, and substantial healthcare costs [8, 24], [30]. The initial stages of biofilm establishment are strongly influenced by the physicochemical properties of the material surface, including surface charge, hydrophobicity, roughness, topography, and chemical composition, which collectively modulate bacterial attachment and maturation of the biofilm structure (Khan and Shakoor [12], [20]. Once implanted, medical devices become part of a complex biological environment in which host cells and microorganisms compete for surface colonization, increasing the risk of device-associated infections [19], [28].

Among the materials used for biomedical applications, titanium and its alloys are particularly relevant due to their excellent mechanical properties, corrosion resistance, and high biocompatibility, which have led to their extensive use in orthopedic and dental implants. Despite these advantages, titanium surfaces remain susceptible to microbial colonization and biofilm development, which can compromise implant function and lead to severe clinical complications [20], Khan and Shakoor [12].

*Pseudomonas aeruginosa* is a clinically important opportunistic pathogen and a well-recognized biofilm former, frequently associated with persistent and difficult-to-treat infections. Its capacity to form dense, structured biofilms on abiotic surfaces, including implant materials, contributes to its high resistance to antibiotics and host immune responses, making infections caused by this organism particularly challenging to eradicate [4, 20].

Current strategies to control biofilm-associated infections rely mainly on conventional antimicrobial therapies and surface modifications; however, these approaches often show limited efficacy against established biofilms and may contribute to the emergence of antimicrobial resistance. This has driven the search for alternative or complementary strategies capable of more effectively targeting biofilm-associated bacteria [6, 11, 21].

In this context, bacteriophages (phages) have re-emerged as promising antibiofilm agents due to their ability to specifically infect bacteria, replicate at the site of infection, and promote bacterial lysis. In addition to direct killing, phages can contribute to biofilm disruption through enzymatic activities that degrade components of the extracellular matrix or by lysing bacteria before or after surface colonization. Moreover, phage cocktails composed of multiple phages with different host ranges have been shown to enhance antibacterial efficacy, resulting in greater reductions in bacterial density in vitro compared to single-phage approaches [13, 14, 16].

Despite these advances, there is still limited quantitative information regarding the effectiveness of hages in both preventing biofilm formation and eradicating mature *P. aeruginosa* biofilms specifically on titanium surfaces, which are highly relevant in clinical practice. Therefore, the present study aimed to evaluate the antibiofilm activity of phages against *P. aeruginosa* on titanium by assessing their ability to reduce established biofilms and to inhibit biofilm formation. By addressing this gap, this work seeks to provide a clearer experimental basis for the development of phage-based strategies targeting biofilm-associated infections on titanium biomedical devices.

## Materials and methods

### Bacterial strains and phages

*Pseudomonas aeruginosa* strain ATCC® 27853™ and *Pseudomonas aeruginosa* clinical strains were provided by the Polydoro Ernani de São Thiago University Hospital, Federal University of Santa Catarina (HU/UFSC). Native lytic phages designated Phage PA1, PA2, PA3, PA4, and PA5 were isolated from clinical *P. aeruginosa* strains and are maintained at the Laboratory of Applied Virology (LVA/UFSC). These five phages were tested for their lytic activity against *P. aeruginosa* using the double agar layer method, and demonstrated effective lytic profiles. After isolation and purification, the phages were propagated using *P. aeruginosa* ATCC strains as host bacteria for amplification and stock preparation. [2], [15].

### Phage treatment of established biofilm

To induce biofilm formation on titanium surfaces, individual assays were performed using *Pseudomonas aeruginosa*. For each test, 20 μL of bacterial inoculum (5 Log CFU/mL) was added to a 24-well microplate containing 980 μL of Brain Heart Infusion (BHI) broth. Titanium elements were immersed in the bacterial suspension using sterile forceps and incubated at 37 °C for 4 days, following a protocol adapted from Freitas et al. [9]. A negative control consisting of titanium elements immersed in sterile BHI broth without bacterial inoculation was incubated under identical conditions. Every two days, the titanium fragments were aseptically transferred to fresh BHI broth (1 mL) after being washed with sterile saline to remove planktonic cells. All experiments were conducted using technical replicates.

For the disruption (treatment) assays, mature 4-day-old biofilms formed on the titanium disks were first gently washed with sterile saline and then transferred to fresh BHI broth (1 mL) containing phages at the indicated multiplicity of infection (MOI). The phages were therefore applied *after* biofilm establishment, and the disks were incubated under the same conditions to evaluate biofilm disruption. For the prophylactic (prevention) assays, phages were added at the beginning of the experiment, together with the bacterial inoculum, and the titanium disks were incubated for 4 days in a 24-well microplate containing 1 mL of BHI broth. Every two days, the disks were transferred to fresh BHI medium (1 mL) to maintain the cultures and assess the preventive activity of the phages against biofilm formation.

Both the disruption and prophylactic experiments were conducted using three different hage concentrations, expressed as multiplicity of infection (MOI): low (MOI 0.01), intermediate (MOI 0.1), and high (MOI 1).

### Viable cell enumeration

To quantify viable bacteria adhered to treated and untreated specimens, each fragment was removed from the nutrient medium and gently rinsed with sterile saline solution. The biofilm remaining on the metal surface was collected using a sterile swab, which was then immersed in a tube containing 2 mL of sterile distilled water. The swab was vortexed for 2 min to detach bacterial cells. The samples were subjected to sonication prior to viability counting in order to al 1 min to ensure thorough cell detachment.

Briefly, 10 μL of the resulting suspension was serially diluted in phosphate-buffered saline (PBS) using base-10 dilutions in 96-well microplates, ranging from 10⁻2 to 10⁻1⁰, to obtain countable CFU (3–30 colonies/mm^2^). Aliquots of 10 μL from each dilution were then vertically dropped onto BHI agar plates. The plates were left open in a laminar flow cabinet to allow the droplets to dry, followed by incubation at 37 °C for 24 h. 

### Assessment by scanning electron microscopy (SEM)

The evaluation of titanium specimens, both with and without biofilms and with or without phage treatment, was performed using Scanning Electron Microscopy (SEM) at the Central Electronic Microscopy Laboratory (LCME/UFSC). Samples were prepared following biofilm cultivation and phage treatment protocols prior to analysis.

Sample preparation for SEM was based on the method recommended by Castro (2002), with modifications. After removal from the suspension, fragments were washed in sterile saline for 1 min and then fixed in 2.5% glutaraldehyde for at least 12 h. Following fixation, samples were dehydrated through a graded ethanol series (30%, 50%, 70%, 90%, and 100%), with each step lasting 30 min. After dehydration, the specimens were immersed in 1% osmium tetroxide for 1 h.

Chemical drying was performed using hexamethyldisilazane (HMDS). Samples were then mounted on aluminum stubs using double-sided conductive adhesive tape and sputter-coated with a 10 nm layer of gold using a Bal-Tec SDC 050 sputter coater for 2 min. Finally, specimens were observed under the SEM at magnifications of 100 × , 1,000 × , and 10,000 × to capture detailed images.

For semi-quantitative analysis of biofilm reduction on the surfaces of titanium elements, the ImageJ software version 1.49. For each experimental group, 5 digital images were used, totaling 50 images, referring to the five phages used, in the three doses applied, and the two strains of *P. aeruginosa*.

Therefore, SEM images and ImageJ-derived parameters were used primarily to support qualitative and semi-quantitative comparisons of biofilm structure and surface coverage, while the quantitative assessment of antibacterial efficacy was based on viability counts.The images were standardized to maintain an adequate scale, with the intention of reducing uncertainties and increasing the precision of the data obtained.

Estimates of the percentage of total area occupied by the biofilm (in black) and the exposed disc (in white) were determined. The images were processed and saved in the TIFF format (Tagged Image File Format), a format commonly used in image binarization and analysis processes. The percentage values obtained were used to calculate the percentage reduction, which is the difference between the initial value (untreated element) and the new value (treated element), a-b, divided by the initial absolute value (untreated element).

### Statistical analysis

The all experiments were performed using technical replicates. Data were analyzed using two-way analysis of variance (Two-Way ANOVA), after verification of the assumptions of normality and homoscedasticity. Normality of the data was assessed using the Shapiro–Wilk test. The level of statistical significance was set at *p* < 0.05. When appropriate, Tukey’s post hoc test was applied for multiple comparisons. All statistical analyses were performed using GraphPad Prism 8.0 software.

## Results

### P. aeruginosa biofilm on titanium

**Fig. 1:**
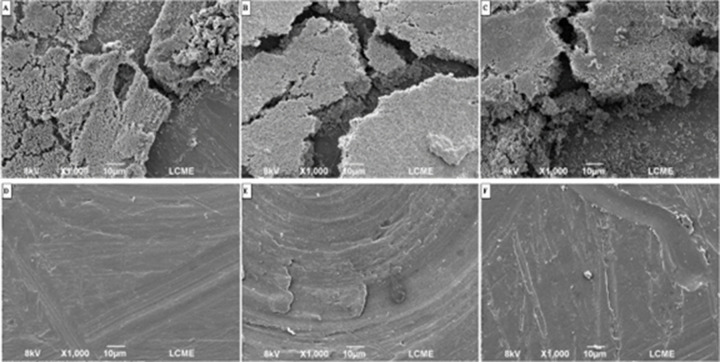
Micrograph of experimental controls: *Pseudomonas aeruginosa* (hospital clinical isolated). Where A, B and C = Positive control, bacterial biofilm adhered to the surface of the specimens; D, E, and F = Negative control, surface of the titanium discs subjected to the same cultivation conditions, but without bacterial inoculum. Both micrographs were taken at the same location, considered the central area of the surface of the specimen—1,000 × magnification

**Fig. 2:**
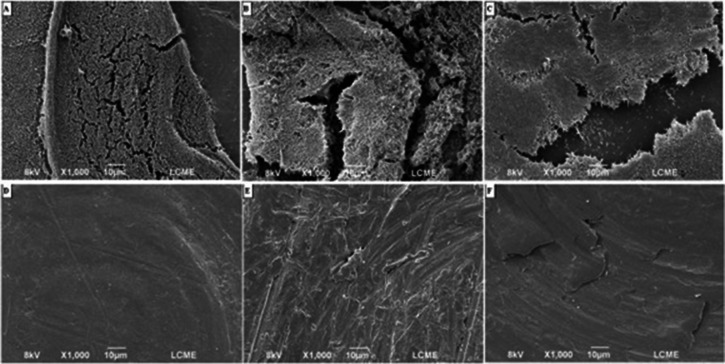
Micrograph of experimental controls: *Pseudomonas aeruginosa* ATCC® 27853™. Where, A, Band C = Positive control, bacterial biofilm adhered to the surface of the specimens; D, E and F = Negative control, surface of the titanium discs subjected to the same cultivation conditions, but without bacterial inoculum. Both micrographs were taken at the same location considered the central area of the surface of the specimen—1,000 × magnification

The SEM images (Figs. 1 and 2) illustrate the surface morphology of titanium elements covered by biofilms formed by *P. aeruginosa* (positive control) compared to titanium discs without biofilm formation (negative control). The titanium surface exhibits notable irregularities, with its rough texture clearly visible. In the positive control images, a dense accumulation of cellular biomass is evident, highlighting extensive biofilm development on the surface.

### Phage-mediated biofilm control: proof of concept

Table 1 presents the survival rates of viable bacterial cells following treatment with hages for each tested infection. Phages demonstrated the ability to infect and reduce the number of viable cells (*p* = 0.03); however, complete eradication was not achieved. Additionally, the results did not exhibit a consistent dose-dependent pattern or clear variation between host strains, preventing the establishment of a definitive cause-and-effect relationship.

**Table 1:** Viable cells recovered from *Pseudomonas aeruginosa* biofilm after treatment with phages in mature biofilm on a titanium surface. Where 0.01, 0.1 and 1 correspond to the viral infection multiplicity indices (PFU/CFU)

*Pseudomonas aeruginosa*	Bacteriophages	Concentration (PFU/CFU)	Survival rate (%)
ATCC® 27853™	PA1	0.01	46.70
	PA1	0.1	26.15
	PA1	1	35.06
	PA2	0.01	28.90
	PA2	0.1	55.42
	PA2	1	8.62
	PA3	0.01	20.30
	PA3	0.1	62.31
	PA3	1	8.84
	PA4	0.01	27.83
	PA4	0.1	37.10
	PA4	1	18.15
	PA5	0.01	61.63
	PA5	0.1	8.84
	PA5	1	26.53
CLINIC	PA1	0.01	3.25
	PA1	0.1	3.20
	PA1	1	7.84
	PA2	0.01	8.81
	PA2	0.1	46.29
	PA2	1	0.22
	PA3	0.01	69.23
	PA3	0.1	76.95
	PA3	1	81.20
	PA4	0.01	31.63
	PA4	0.1	34.27
	PA4	1	53.79
	PA5	0.01	12.41
	PA5	0.1	61.53
	PA5	1	33.82

**Fig. 3:**
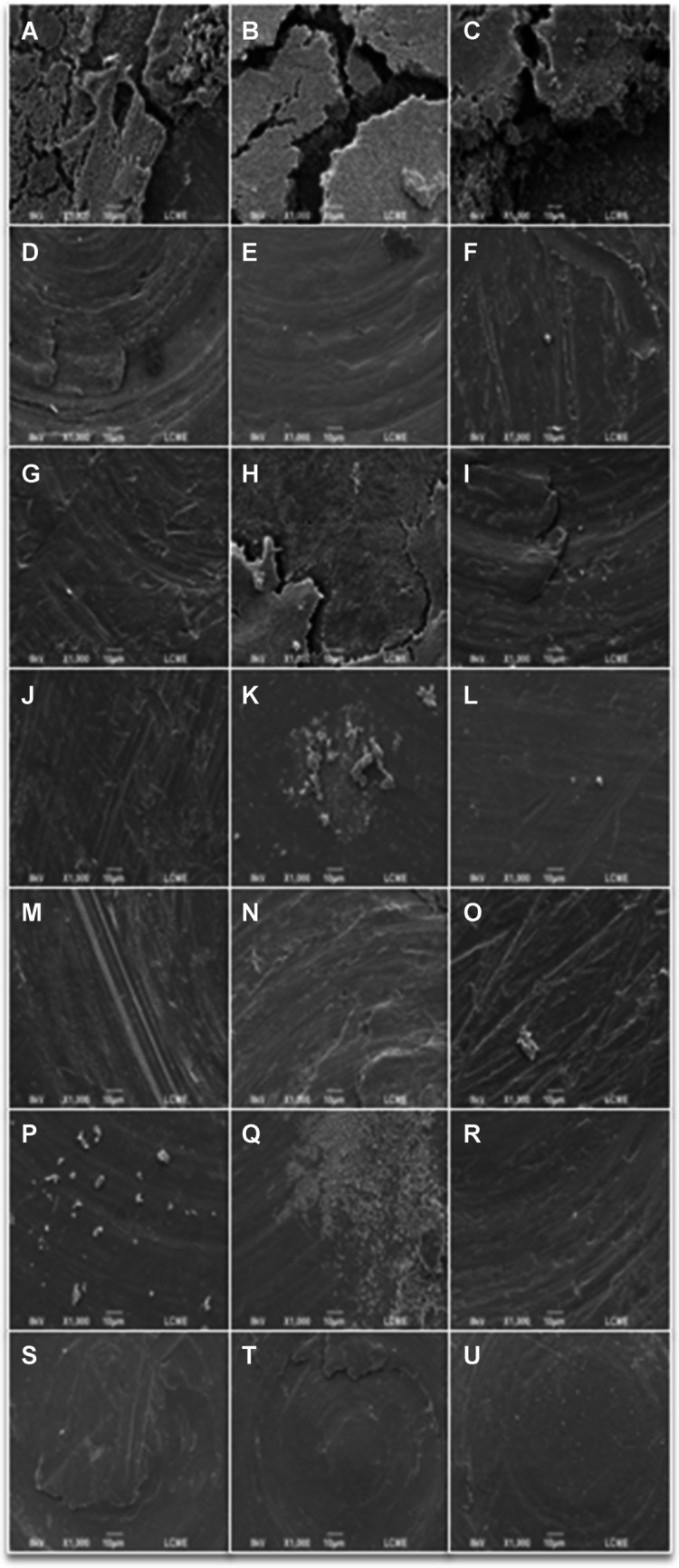
Scanning electron microscopy (SEM) evaluation of the disaggregation and destructive activity of phage treatment against clinical *Pseudomonas aeruginosa* biofilms formed on titanium surfaces. Panels A–C correspond to the positive control (untreated biofilm), and panels D–F correspond to the negative control. Controls were generated from the same experimental batch and processed under identical fixation and preparation conditions as the treated samples. Panels G–U represent phage-treated samples. Rows correspond to the specific phages tested: PA1 (G–I), PA2 (J–L), PA5 (M–O), PA3 (P–R), and PA4 (S–U). Columns indicate increasing multiplicities of infection (MOI): MOI 0.01 (G, J, M, P, S), MOI 0.1 (H, K, N, Q, T), and MOI 1 (I, L, O, R, U). All micrographs were obtained from the central region of the titanium specimen surface at a magnification of 1,000 ×

The micrographs presented in Fig. 3 show the reduction in biofilm of the *P. aeruginosa* clinical strain on the surface of the titanium elements after treatment with the 5 phages tested, compared to the control. Regarding phages PA1, PA2, and PA3, the intermediate dose was less efficient in eradicating biofilm compared to the other concentrations applied (*p* = 0.04). Among these, PA3 was the least efficient at the lowest dose. The PA4, on the other hand, was considered the most efficient, as there was no presence of post-treatment biofilm at either concentration, as was the case with PA5; however, traces of biofilm were observed at the highest concentration tested.

**Fig. 4:**
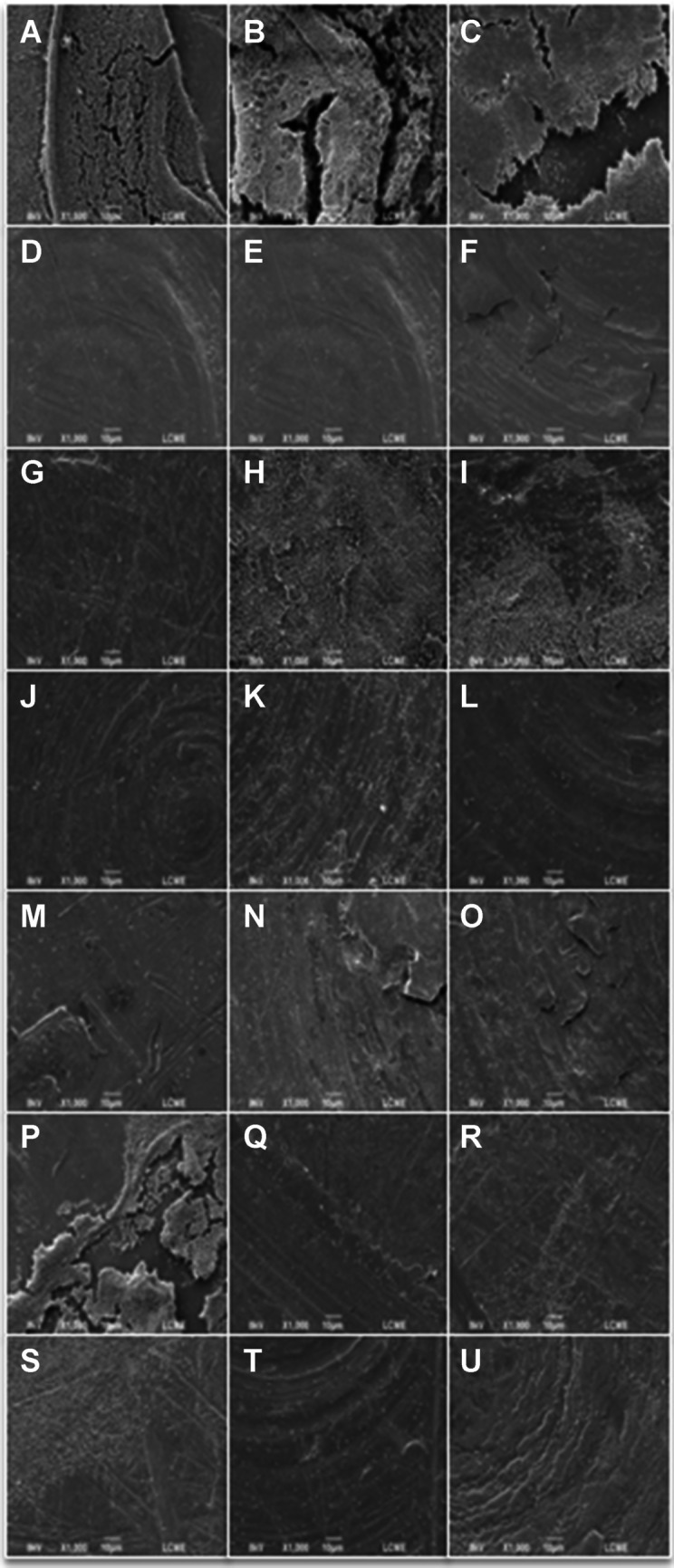
Figure 4 Scanning electron microscopy (SEM) evaluation of the disaggregation and destructive activity of phage treatment against *Pseudomonas aeruginosa* ATCC® 27853™ biofilms formed on titanium surfaces. Panels A–C correspond to the positive control (untreated biofilm), and panels D–F correspond to the negative control. Controls were generated from the same experimental batch and processed under identical fixation and preparation conditions as the treated samples.Panels G–U represent phage-treated samples. Rows correspond to the specific phages tested: PA1 (G–I), PA2 (J–L), PA5 (M–O), PA3 (P–R), and PA4 (S–U). Columns indicate increasing multiplicities of infection (MOI): MOI 0.01 (G, J, M, P, S), MOI 0.1 (H, K, N, Q, T), and MOI 1 (I, L, O, R, U). All micrographs were obtained from the central region of the titanium specimen surface at a magnification of 1,000 ×

The same general observation regarding the disaggregation capacity was noted for *P. aeruginosa* ATCC strain (Fig. 4). However, there was a difference regarding the profile. The PA3 and PA4 phages had the lowest efficiency, allowing for the visualization of biofilm and a high bacterial population density, respectively. The intermediate and high doses were effective in both (*p* = 0.04), except for PA2, where planktonic cells were observed in abundance. However, when comparing the intermediate and high dosages of PA1 phage, the high dosage was more efficient.

### Phage-mediated biofilm control quantify

Table 2 presents the reduction of *P. aeruginosa* biofilm per total surface area (mm2) of the titanium piece. For the ATCC strain, the PA1 phage showed a biofilm removal efficiency range of 50.53% to 99.78%; PA2, greater than 99%; PA3, between 71.60% and 99.16%; PA4, 87.55% to 99.70%; and PA5, also higher than 99%. Considering clinical strain, PA1 showed a reduction rate between 0.01% and 99.82%; PA2, between 92.61% and 99.78%; PA3, between 75.31% and 99.94%; PA4, greater than 99%; and finally, PA5, greater than 90%. Therefore, there is efficiency in the application of phages for the destruction and disintegration of *P. aeruginosa* biofilm (*p* = 0.03).

**Table 2:** Reduction of *Pseudomonas aeruginosa* biofilm on the surface of titanium elements by Scanning Electron Microscopy (SEM). Software for editing and analyzing micrographs, ImageJ © version 1.49, using the Java language

*Pseudomonas aeruginosa*	Bacteriophage (MOI)	MOI	Micrograph area (%)	Disc biofilm area mm^2^ (control)	Disc biofilm area mm^2^ (treatment)	Reduction rate (%)
ATCC® 27853™	PA1	0.01	0.202	17.69	0.040	98.78
	PA1	0.1	44.557		8.751	50.53
	PA1	1.0	31.012		6.091	65.57
	PA2	0.01	0.004		0.001	100
	PA2	0.1	0.519		0.116	99.34
	PA2	1.0	0.077		0.015	99.91
	PA3	0.01	25.579		5.024	71.60
	PA3	0.1	0.761		0.149	99.16
	PA3	1.0	3.143		0.617	96.51
	PA4	0.01	11.216		2.203	87.55
	PA4	0.1	0.563		0.111	99.37
	PA4	1.0	0.273		0.054	99.70
	PA5	0.01	0.147		0.029	99.84
	PA5	0.1	0.45		0.088	99.50
	PA5	1.0	0.162		0.032	99.82
CLINIC	PA1	0.01	0.196	16.66	0.038	99.77
	PA1	0.1	84.81		16.657	0.01
	PA1	1.0	0.188		0.037	99.78
	PA2	0.01	0.253		0.050	99.70
	PA2	0.1	6.269		1.231	92.61
	PA2	1.0	0.125		0.025	99.85
	PA3	0.01	2.084		0.409	97.54
	PA3	0.1	20.942		4.113	75.31
	PA3	1.0	0.048		0.009	99.94
	PA4	0.01	0.209		0.041	99.75
	PA4	0.1	0.104		0.020	99.88
	PA4	1.0	0.052		0.010	99.94
	PA5	0.01	6.839		1.347	91.91
	PA5	0.1	0.01		0.002	99.99
	PA5	1.0	0.188		0.37	99.78

### Phage-mediated biofilm control: prophylaxis assay

#### Bacteria viable count

Table 3 presents the bacterial survival after biofilm formation and subsequent prophylactic treatment with phages for both the clinical and reference strains. The data indicate that the phages effectively reduced the number of viable bacterial cells, resulting in strong suppression of biofilm-associated bacteria, with survival rates typically below 3%. No clear dose–response relationship was observed across the tested multiplicities of infection, and variability between strains was noted, highlighting the complexity of host–phage interactions and making it difficult to establish a strict cause-and-effect relationship.

**Table 3:** Viable cells recovered from *Pseudomonas aeruginosa* biofilm on a titanium surface after prophylactic treatment with phages at different concentrations. Where 0.01, 0.1, and 1 correspond to the viral infection multiplicity indices (UFP/UFC). UFC = Colony Forming Unit per mL; UFP Unit of plaque per mL

*Pseudomonas aeruginosa*	Bacteriophages	MOI	Survival rate (%)
ATCC® 27853™	PA1	0.01	0.19
	PA1	0.1	0.07
	PA1	1	0.14
	PA2	0.01	0.15
	PA2	0.1	0.52
	PA2	1	0.3
	PA3	0.01	0.09
	PA3	0.1	0.08
	PA3	1	0.61
	PA4	0.01	0.15
	PA4	0.1	0.40
	PA4	1	0.10
	PA5	0.01	0.67
	PA5	0.1	2.51
	PA5	1	1.69
CLINIC	PA1	0.01	0.05
	PA1	0.1	0.15
	PA1	1	0.04
	PA2	0.01	2.92
	PA2	0.1	0.41
	PA2	1	0.01
	PA3	0.01	0.23
	PA3	0.1	0.16
	PA3	1	0.26
	PA4	0.01	1.39
	PA4	0.1	0.45
	PA4	1	0.11
	PA5	0.01	0.07
	PA5	0.1	0.21
	PA5	1	0.34

**Fig. 5:**
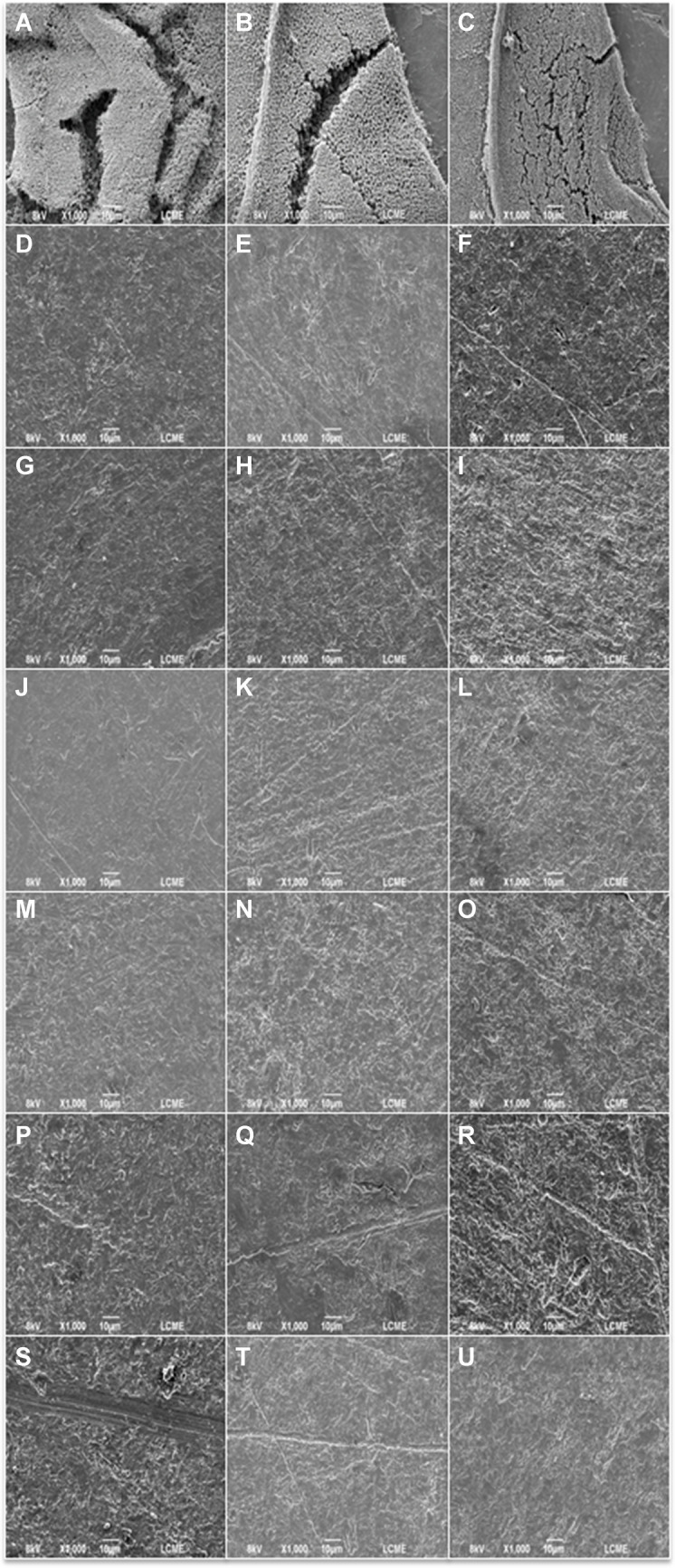
Scanning electron microscopy (SEM) evaluation of the prophylactic activity of phage treatment during the formation of *Pseudomonas aeruginosa* ATCC® 27853™ biofilm on titanium surfaces. Panels A–C correspond to the positive control (biofilm formation in the absence of phage), and panels D–F correspond to the negative control. Controls were generated from the same experimental batch and processed under identical fixation and preparation conditions as the treated samples. Panels G–U represent phage-treated samples. Rows correspond to the specific phages tested: PA1 (G–I), PA2 (J–L), PA5 (M–O), PA3 (P–R), and PA4 (S–U). Columns indicate increasing multiplicities of infection (MOI): MOI 0.01 (G, J, M, P, S), MOI 0.1 (H, K, N, Q, T), and MOI 1 (I, L, O, R, U). All micrographs were obtained from the central region of the titanium specimen surface at a magnification of 1,000 ×

**Fig. 6:**
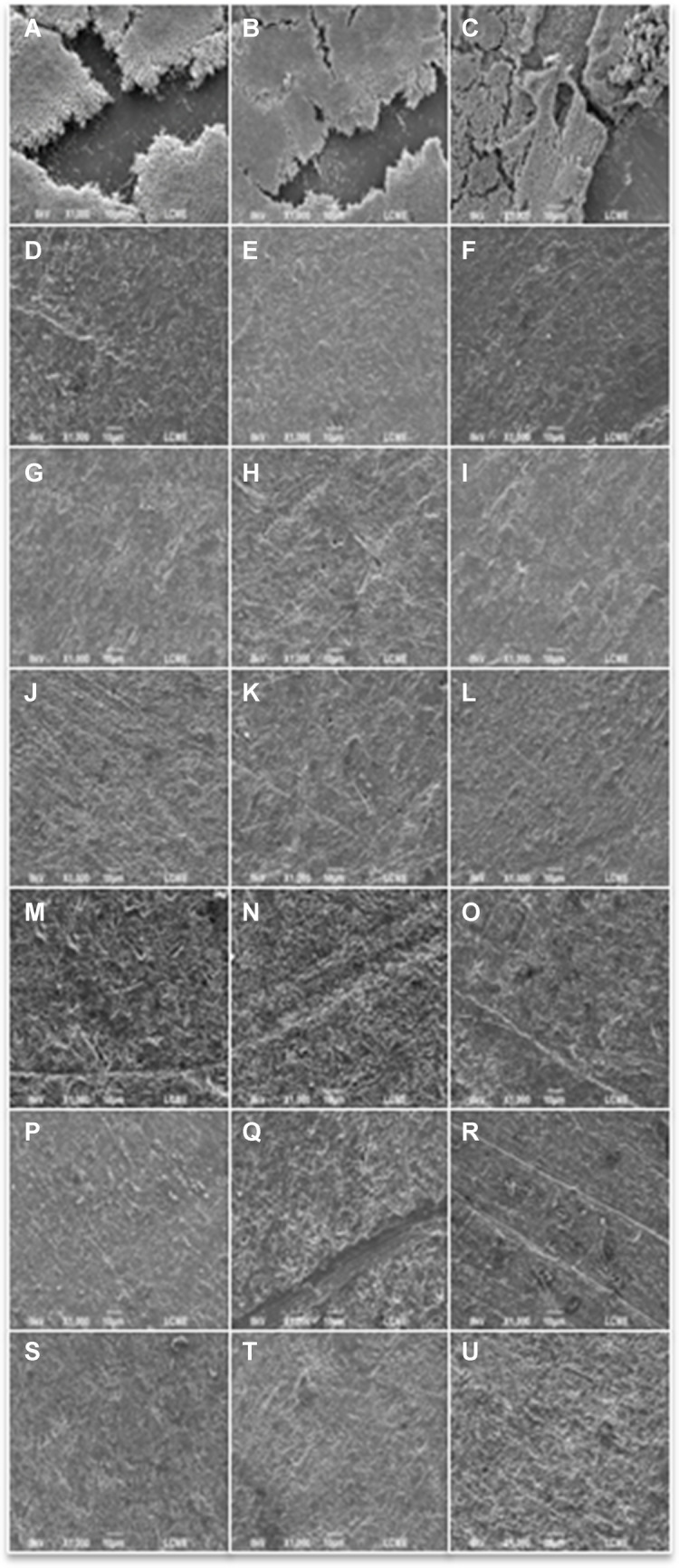
Evaluation of the prophylactic activity of phage treatment during the formation of clinical *Pseudomonas aeruginosa* biofilm on titanium surfaces. Panels A–C correspond to the positive control (biofilm formation without phage), and panels D–F correspond to the negative control. Controls were generated from the same experimental set and processed simultaneously with the treated samples. Panels G–U represent phage-treated specimens arranged by rows according to the phage tested: PA1 (G–I), PA2 (J–L), PA5 (M–O), PA3 (P–R), and PA4 (S–U). Columns indicate increasing MOI: MOI 0.01 (G, J, M, P, S), MOI 0.1 (H, K, N, Q, T), and MOI 1 (I, L, O, R, U). All micrographs were obtained from the central region of the titanium surface at a magnification of 1,000 ×

In Fig. 5, micrographs of the titanium specimen surface obtained through SEM are presented. The SEM images show prophylactic activity with 100% inhibition of biofilm formation in the observed area, without variation between the study variables (concentration and host–pathogen interaction). The same can be found in Fig. 6, for tests with the clinical strain.

Considering the percentage reduction of *P. aeruginosa* biofilm on the surface of titanium elements measured by Scanning Electron Microscopy (SEM) after prophylactic treatment and calculated per surface area (mm^2^), an efficiency of 100% was observed for all phages and concentrations tested. These results indicate complete inhibition of biofilm formation, demonstrating the total efficacy of phage application as a preventive strategy against *P. aeruginosa* biofilm development on titanium surfaces.

## Discussion

This study demonstrated the effective use of phages in controlling *Pseudomonas aeruginosa* biofilms on titanium surfaces. There are several notable findings in the study: (i) The possible multifage collections used were able to infect and reduce viable bacterial cells, making the pathogen more susceptible; (ii) the efficacy of phages in reducing viable cells in the biofilm is highly dependent on the specificity of the phage, bacterial strain, and treatment concentration; (iii) analysis of micrographs showed that phages are capable of reducing *P. aeruginosa* biofilms on titanium discs by over 90%; (iv) When used prophylactically, phages inhibited 100% of biofilm formation.

Strategies for combating bacterial biofilms are divided into two segments: eradicating already formed biofilms or inhibiting their formation. Among the strategies for biofilm inhibition, inhibiting bacterial growth using bactericidal or bacteriostatic compounds, as well as blocking bacterial adhesion, are considered. Antivirulence therapies are also seen as an alternative, as they make the pathogen more susceptible to the immune system and antimicrobials [5], [23].

The findings in this study also demonstrate the efficacy of phages in inhibiting biofilm formation and disintegration of already formed biofilm, as well as their activity in reducing the growth rate of *P. aeruginosa*. The phage can also indirectly destroy biofilms either through its enzymatic activity or by causing bacterial lysis before or after colonization of the surface [7], [27] .

The use of phage cocktails containing two or more phage mixtures with different host ranges in a single suspension has been shown to be more effective in inhibiting bacterial infections. As in the study by Kovacs et al. [14], the multifage collection resulted in better reduction of bacterial density in vitro, enhancing the efficacy of phages. Unlike antibiotics used for such purposes, phages have greater ease of penetration into *P. aeruginosa* biofilm; the enzymatic activity of phages, as portrayed in various studies, aids in the adsorption, invasion, and disintegration of the host bacteria. Additionally, phages produce endolysins capable of degrading peptidoglycans and assisting in the formation of new progeny phages for release and initiation of a new cycle of viral infection [13, 14, 16, 17]

Therefore, the advantages of using phages compared to antibiotics in inhibiting infections caused by bacterial biofilms are evident due to their high specificity and unique mode of action against sessile cells, such as their ability to infect and destroy persistent cells upon reactivation. Another important mechanism is their ability to dissolve the extracellular polymeric matrix through the production of their own enzymes or induction of enzymatic production by the host organism, as well as through interruption of quorum-sensing communication [1, 14].

Several studies have highlighted the advances in phage therapy against clinically important bacteria in the clinical environment, such as *P. aeruginosa*, which causes diseases like chronic otitis, cystic fibrosis, and burns. However, these studies have been limited by preclinical evaluations. Despite the absence of legislation in Brazil authorizing and regulating the use of phage therapy, it should be noted that phage therapy is not a new approach, having been widely used in Brazil in 1920 by José da Costa Cruz of the Oswaldo Cruz Institute (Rio de Janeiro, Brazil) in the control and treatment of bacillary dysentery and staphylococcal infections Almeida and Sundberg [3].

*In vitro*, *in silico*, and *in vivo* studies have been conducted, indicating the safety and efficacy of phages in controlling major clinical gram-negative and gram-positive pathogens, especially those microorganisms with high resistance to antibiotics, high morbidity and mortality, and high biofilm production. Kutateladze and Adamia [18] suggested that phage therapy can be used in biocontrol in medical and dental devices, hospital environments, and mainly in the treatment of nosocomial infections in humans and animals (Hampton et al. [10], [18, 26, 29].

## Conclusion

In conclusion, this study demonstrates that phages can effectively reduce and inhibit *Pseudomonas aeruginosa* biofilms on titanium surfaces under in vitro conditions. The results, supported by CFU counts and semi-quantitative SEM observations, indicate that phage treatment can decrease viable bacterial cells and disrupt biofilm structure, while the use of phage cocktails and consideration of bacterial strain specificity and phage concentration emerge as important factors influencing efficacy. Although these findings are promising, the mechanisms underlying biofilm reduction such as bacterial lysis and enzymatic degradation of the extracellular matrix were inferred from literature rather than directly measured. Methodological limitations, including the use of technical replicates and in vitro models, are acknowledged. Consequently, the translational relevance of this work is prospective: further in vivo and clinical studies are required to optimize phage therapy, evaluate its safety and effectiveness, and advance its potential application in controlling biofilm-associated infections on biomedical devices.

## Data Availability

No datasets were generated or analysed during the current study.
